# Precision medicine for Parkinson’s disease: The subtyping challenge

**DOI:** 10.3389/fnagi.2022.1064057

**Published:** 2022-12-01

**Authors:** Mark Frasier, Brian K. Fiske, Todd B. Sherer

**Affiliations:** The Michael J. Fox Foundation for Parkinson’s Research, New York, NY, United States

**Keywords:** Parkinson’s disease, drug development, biomarkers, subtypes, staging, precision medicine

## Abstract

Despite many pharmacological and surgical treatments addressing the symptoms of Parkinson’s disease, there are no approved treatments that slow disease progression. Genetic discoveries in the last 20 years have increased our understanding of the molecular contributors to Parkinson’s pathophysiology, uncovered many druggable targets and pathways, and increased investment in treatments that might slow or stop the disease process. Longitudinal, observational studies are dissecting Parkinson’s disease heterogeneity and illuminating the importance of molecularly defined subtypes more likely to respond to targeted interventions. Indeed, clinical and pathological differences seen within and across carriers of PD-associated gene mutations suggest the existence of greater biological complexity than previously appreciated and increase the likelihood that targeted interventions based on molecular characteristics will be beneficial. This article offers our current perspective on the promise and current challenges in subtype identification and precision medicine approaches in Parkinson’s disease.

## Introduction

Death of nigrostriatal dopaminergic neurons is a pathological hallmark of Parkinson’s disease (PD). Indeed, most approved PD therapies today seek to replace or mimic dopamine’s molecular activity. While remarkably effective in treating many of the disease’s troubling motor symptoms, the efficacy of dopaminergic drugs becomes more complicated over time as dopamine neurons continue to degenerate. Moreover, degeneration in other non-dopaminergic systems and emergence of non-motor or treatment-resistant motor symptoms such as cognitive impairment, dementia, autonomic dysfunction, and gait impairment, makes management of PD, especially in later stages, more challenging. Despite tests of interventions to potentially slow disease progression (McFarthing et al., 2022), there are no approved therapies that modify the underlying pathophysiological causes of PD’s neurodegenerative process.

The central role of dopamine has fueled a long-held perception that PD is a *single disease*. However, this idea ignores the fact that research, including human genetic studies, suggests a variety of molecular mechanisms as cellular culprits behind PD neurodegeneration including neuroinflammation, lysosomal dysfunction, and disruption of mitochondrial pathways (Jankovic and Tan, 2020). Environmental exposures to factors including pesticides and heavy metals may also contribute to some forms of PD. Recent imaging data also suggests the possibility of ‘brain-first’ and ‘body-first’ progression of PD depending on observed pathology and clinical symptoms (Horsager et al., 2020). Clinical heterogeneity also exists in the presentation and progression of PD symptoms (Lee et al., 2022). The combination of this biological and clinical variability, and the implication that it reflects more complex disease subtypes, hampers accurate prognosis and treatment strategies for people with PD.

We believe successful development of therapies that slow or prevent neuronal loss and subsequent disease progression in PD requires a more precise understanding and subsequent targeting of the molecular causes of PD, an idea that we and others have raised often (Sherer et al., 2016; Sturchio et al., 2020; von Linstow et al., 2020; Schalkamp et al., 2022). To do this, we will need to establish molecular-based definitions of Parkinson’s subgroups. By leveraging a growing number of shared datasets along with computational analyses to expand our understanding of PD and its subtypes, the PD research community has an unprecedented opportunity to accelerate progress toward meaningful and impactful new treatments. At The Michael J Fox Foundation for Parkinson’s Research, our strategic priorities have supported deep data collection from well-characterized and diverse cohorts and funded analyses to explore and validate putative disease subtypes. By harnessing historical and emerging advances and data from genetic and biomarker research, we think the potential for more tailored therapeutic approaches is within reach.

## Early clinical phenotyping

Initial attempts to categorize PD into subtypes relied on neurologists’ acute but infrequent observations of Parkinson’s symptoms over multiple visits. While PD is traditionally characterized by cardinal symptoms—tremor, bradykinesia, and rigidity—movement disorder specialists often described clinical features suggestive of at least two subtypes within PD: individuals who experience tremor as the most significant movement symptom (tremor dominant) and individuals who exhibit bradykinesia, postural instability and gait disturbances (posture gait instability disorder) (Zetusky et al., 1985). This grouping pointed to the idea that Parkinson’s disease could be a syndrome comprising several distinct disorders harboring unique clinical and pathophysiological mechanisms. However, later research revealed that reliance on clinical subtypes may be insufficient in describing the true heterogeneity of PD. When newly diagnosed and yet untreated people with PD were followed longitudinally, there was significant instability in the subtypes: patients appeared to ‘switch’ between tremor dominant and posture gait instability disorder forms and vice versa in the early phases of disease (Simuni et al., 2016). Other studies suggest that PD subtypes are unlikely to be binary. If researchers integrate assessments of nonmotor symptoms such as sleep, mood, and cognition, the number of subtype clusters increases to five, with differences mainly driven by the symptoms not responsive to dopaminergic medication (Lawton et al., 2015). Most telling perhaps, a comprehensive review highlighted deficiencies and lack of replication as a significant barrier to clinical validation of subtypes (Mestre et al., 2021). It is clear, then, that describing the phenotype of PD using clinical features *alone* is insufficient to describe the many forms PD can take. Other data modalities including neuroimaging and molecular data as well as more objective and frequent assessments are therefore needed to adequately define PD.

## The emergence of genetic phenotyping

Genetic studies of PD over the last 20 years have revolutionized our understanding of the causes and contributors to PD and identified druggable molecular targets. Analyses of families with a history of PD uncovered monogenic, presumably causal mutations (Ferreira and Massano, 2017). Inclusion of data from genomewide association studies adds, as of now, an additional 90 genetic loci conferring increased risk for Parkinson’s disease (Nalls et al., 2019). These insights have been fundamental in fueling investment in drug development programs targeting proteins encoded by several of the associated genes, with multiple drug makers prioritizing therapies targeting genetic contributors such as alpha-synuclein, glucocerebrosidase, and LRRK2.

Longitudinal observation of individuals carrying different mutations suggest that clinical features may cluster with certain mutations, offering an additional layer of precision. People with PD carrying LRRK2 mutations tend to progress slower in their motor symptoms over time, while individuals harboring GBA mutations are more likely to develop cognitive impairment (Smith et al., 2022). Carriers of mutations in or multiplication of SNCA, the gene encoding the alpha-synuclein protein, have a more rapid progression including significant nonmotor features, while carriers of mutations in PRKN, encoding the parkin protein, manifest a ‘purer’ motor symptom phenotype (Tambasco et al., 2016; Book et al., 2018). Postmortem examination of brains representing a range of gene mutation carriers also reveals differences in pathological features: GBA mutation carriers frequently exhibit the presence of Lewy bodies in striatal and cortical regions, while a subset of LRRK2 mutation carriers harbor phosphorylated tau tangles without evidence of Lewy pathology (Smith et al., 2022). Parkin mutation carriers typically show only dopamine neuron degeneration with no accompanying Lewy pathology or other cell loss (Wasner et al., 2020). Taken together, clinical and pathological differences within and across PD gene carriers provide evidence for the existence of subtypes and suggest that a more targeted therapeutic strategy may be warranted.

This idea is already being pursued in clinical trials as sponsors look to enroll participants based on genetic status, hoping that individuals harboring mutations in specific genes may be most responsive to targeted treatments (von Linstow et al., 2020). In the case of GBA and LRRK2, two of the most prevalent genetic forms of PD, sufficient numbers of people with PD carrying mutations in these genes exist such that multi-site clinical trials can be designed focused solely on enrolling mutation carriers. Evidence of this approach can be seen in a recent trial testing venglustat, an inhibitor of glucosylceramide synthase, in people with PD harboring GBA mutations (Peterschmitt et al., 2022). While the trial ultimately failed to meet its endpoints, the study represented an early proof case for successful enrichment of a specific PD genetic subtype for a therapeutic trial. Similarly, an inhibitor of LRRK2 kinase activity is being tested in PD carriers of the LRRK2 G2019S mutation, which appears to abnormally elevate LRRK2’s kinase’ activity (NIH National Library of Medicine, 2022a,b). Finally, a trial of a cell replacement transplantation strategy is enrolling people with PD carrying mutations in the gene for parkin, hypothesizing these individuals will benefit most since PD caused by parkin mutations appear to be more purely linked to dopaminergic cell loss (The Michael J. Fox Foundation, 2022).

In all trials enrolling individuals based on the presence of a genetic variant, it will be important to consider variability in clinical phenotype of mutation carriers, even within the same gene. Deeper interrogation of different mutations within the LRRK2 gene reveals a more complex picture where the clinical phenotype may vary based on the specific mutation (Goveas et al., 2021). Understanding the biologic impact and downstream molecular consequences of the different mutations will likely guide different therapeutic strategies. This includes both rare and more frequently occurring mutations associated with Parkinson’s disease and parkinsonisms, since these may share similar biologic underpinnings resulting in disparate phenotypes. Interventions targeting Parkinson’s-associated genes could help individuals with Parkinson’s disease that do not carry the genetic variant if they share similar disruptions in molecular pathways. In support of this, LRRK2 inhibitors or anti-sense oligonucleotides might benefit non-LRRK2 carriers since many non-carriers do exhibit increased kinase activity (Cook et al., 2017), and in fact trials are occurring in both mutation carriers and non-carriers (NIH National Library of Medicine, 2022a,b).

## The future of PD subtyping

As the field advances, we expect that the next few years of PD subtyping will leverage additional data modalities including molecular, clinical, neuroimaging, and digital sensor data. The availability of open-access datasets such as Parkinson’s Progression Markers Initiative (n.d.) and Accelerated Medicine Initiative for PD (AMP PD, n.d.) paired with more sophisticated data analysis techniques should further refine subgroups that may benefit from more tailored treatments. Indeed, the application of advanced machine learning approaches have already begun to identify clusters of PD subtypes (Zhang et al., 2019; Severson et al., 2021). Integrating motor and nonmotor clinical data with targeted molecular markers has linked certain blood analytes with clinical features (Lawton et al., 2020). High dimensional molecular data using transcriptomic and proteomic platforms will also drastically increase the ability to map out molecular signatures of PD, and we predict these data will be combined with genetic and clinical features to identify those who may be most responsive to targeted therapies. Finally, capturing the lived experience of people with PD using remote sensor technologies will offer even greater insight into possible subtypes along with methods for assessing impact of targeted interventions.

One molecular measure of suspected pathology is showing particular promise. The alpha-synuclein protein is a key player in Parkinson’s pathophysiology, and measuring all forms of protein (soluble, insoluble, total, post-translationally modified) may uncover more distinct signatures of Parkinson’s subgroups. Assays measuring the alpha-synuclein protein combined with clinical data are yielding intriguing results suggestive of subtypes that may distinguish individuals with presumed high alpha-synuclein pathology burden. Across multiple laboratories and assay platforms, alpha-synuclein seeding amplification assay (SAA) can diagnose individuals with Parkinson’s disease with accuracy of over 95% (Russo et al., 2021). As SAA positivity rate varies depending on the gene and mutation (Brockmann et al., 2021), the assay can be used in combination with genetic status and other clinical features to refine PD subtype understanding. For example, the assay has only 70% positivity in LRRK2 PD patients and the positive subjects appear to manifest olfactory deficits, while the remaining 30% appear to have normal olfaction (Siderowf et al., 2022). Compellingly, in a small number of LRRK2 autopsy cases for which antemortem CSF SAA data exists, a positive result correlates with presence of brain alpha-synuclein pathology, while those that are negative do not appear to harbor alpha-synuclein accumulation in the brain (Siderowf et al., 2022). While this observation needs to be replicated in a larger dataset, these studies are an exciting example of how a fluid measure might identify individuals with high alpha-synuclein pathology burden and could be used as an enrichment tool for clinical trials using a targeted alpha-synuclein therapy.

Combining clinical features with genetics offers an exciting opportunity to identify people at the earliest stages of their clinical journey and explore the molecular changes occurring. Olfaction, constipation, and REM sleep behavior disorder (RBD) can help find at-risk individuals and analysis of DNA will help identify molecular subtypes. As genetic data from people with PD continues to increase, we are also seeing advances in how this genetic insight can be leveraged for stratifying risk not just to PD but to specific biological forms. For example, recent work with polygenic risk scores suggests an ability to tease out not only likelihood of PD but also PD associated with pathway-defined risk, such as mitochondrial or autophagy-lysosomal forms (Dehestani et al., 2022). Advances in understanding these molecular signatures will inform clinical trials on what pathways are likely to be more targetable within a given population. With large-scale efforts ongoing, such as Global Parkinson’s Genetics Program (2021), we will likely see even greater advances in the use of genetic information to guide subtype understanding.

## Discussion

Translating these early molecular indicators of Parkinson’s subtypes into reliable and clinically useful precision medicine tools will require collaboration across the field. Data sharing from both observational studies and clinical trials is essential to validate existing and identify novel disease subtypes. The PPMI, AMP PD, and Critical Path for Parkinson’s Disease (CPP) initiatives are public private consortia built around data sharing of human observational studies and clinical trials with the goal of developing more effective PD treatments. Learning from clinical trials testing therapeutics in enriched PD populations will help the field understand the benefits and risks of targeted treatment strategies in PD. Posthoc analyses of historical trials will reveal features of patients that may have responded more favorable to treatment than others, and this will only improve the stratification of future clinical trials. Encouragingly, the infrastructure for data sharing already exists through these consortia, and a maturing culture among PD researchers is fostering a similar expectation of data sharing and collaboration.

The PD research community also needs to engage regulators in conversations about precision medicine approaches. Both the Food and Drug Administration and European Medicines Agency have indicated the importance of measuring clinical meaningfulness in clinical trials testing novel therapeutics. If precision medicine in PD will make progress, early and frequent input from regulators will be critical to understand the data needed to link PD subtypes and targeted treatment with desired clinical outcomes. Fortunately, initiatives like CPP are established venues in which all stakeholders convene and discuss these challenges.

We also need continued investment in targeted molecular assays focused on measuring the spectrum of PD pathophysiology. The Michael J Fox Foundation for Parkinson’s Research (MJFF) has invested in developing and optimizing imaging and biochemical measurements across hypothesized pathways linked to PD including neuroinflammation, lysosomal dysfunction, and mitochondrial dysfunction. Expanding the toolkit of such assays and linking to data from multiple patient cohorts should help identify subtypes that could benefit from a therapeutic targeting a specific molecular pathway. However, molecular changes clustering together in subgroups may be orthogonal to clinical features of PD and may not correlate with clinical outcomes. This will require a fundamental shift in how the community defines PD and will result in a *biologic staging* of disease based on molecular changes rather than definitions based on pure clinical phenomenology. Indeed, PD will require a consensus molecular staging similar to what has been done for Alzheimer’s disease (Jack et al., 2018).

Finally, we must engage all people affected by PD. Data from people living with the disease will critically fuel precision medicine but will only succeed by enlisting their input throughout the research process from study design to data analysis. As most PD data are derived from people of European Caucasian descent, it will also be essential to partner with a wider and more diverse group of individuals to obtain a full picture of the heterogeneity of PD (and ultimately more precise understanding of disease subtypes).

The Parkinson’s community including patients, researchers, industry sponsors, regulators, and nonprofits is poised to transform the approach to therapeutic development for Parkinson’s disease. An increasing mindset of data sharing and collaboration (and the platforms to do so), advances in molecular and digital data collection, along with data infrastructure and analytic capabilities are the foundation on which precision medicine for Parkinson’s disease will be built. Much like shifts seen in oncology, where the term ‘cancer’ now encompasses a wide spectrum of molecularly defined tumor types, each with selective treatment, we believe that PD will soon follow a similar path.

## Data availability statement

Publicly available datasets were analyzed in this study. This data can be found at: https://www.ppmi-info.org, https://amp-pd.org/.

## Author contributions

All authors listed have made a substantial, direct, and intellectual contribution to the work and approved it for publication.

## Conflict of interest

MF, BF, and TS are employed by The Michael J Fox Foundation for Parkinson’s Research.

## Publisher’s note

All claims expressed in this article are solely those of the authors and do not necessarily represent those of their affiliated organizations, or those of the publisher, the editors and the reviewers. Any product that may be evaluated in this article, or claim that may be made by its manufacturer, is not guaranteed or endorsed by the publisher.
